# Defining the molecular pathology of pancreatic body and tail adenocarcinoma

**DOI:** 10.1002/bjs.10772

**Published:** 2018-01-17

**Authors:** Australian Pancreatic Cancer Genome Initiative, S B Dreyer, N B Jamieson, R Upstill-Goddard, P J Bailey, C J McKay, A V Biankin, D K Chang

**Affiliations:** West of Scotland Pancreatic Unit, Glasgow Royal Infirmary, Glasgow, UK; Institute of Cancer Sciences, University of Glasgow, Glasgow, UK

## Abstract

**Background:**

Pancreatic ductal adenocarcinoma (PDAC) remains a dismal disease, with very little improvement in survival over the past 50 years. Recent large-scale genomic studies have improved understanding of the genomic and transcriptomic landscape of the disease, yet very little is known about molecular heterogeneity according to tumour location in the pancreas; body and tail PDACs especially tend to have a significantly worse prognosis. The aim was to investigate the molecular differences between PDAC of the head and those of the body and tail of the pancreas.

**Methods:**

Detailed correlative analysis of clinicopathological variables, including tumour location, genomic and transcriptomic data, was performed using the Australian Pancreatic Cancer Genome Initiative (APGI) cohort, part of the International Cancer Genome Consortium study.

**Results:**

Clinicopathological data were available for 518 patients recruited to the APGI, of whom 421 underwent genomic analyses; 179 of these patients underwent whole-genome and 96 RNA sequencing. Patients with tumours of the body and tail had significantly worse survival than those with pancreatic head tumours (12·1 *versus* 22·0 months; *P* = 0·001). Location in the body and tail was associated with the squamous subtype of PDAC. Body and tail PDACs enriched for gene programmes involved in tumour invasion and epithelial-to-mesenchymal transition, as well as features of poor antitumour immune response. Whether this is due to a molecular predisposition from the outset, or reflects a later time point on the tumour molecular clock, requires further investigation using well designed prospective studies in pancreatic cancer.

**Conclusion:**

PDACs of the body and tail demonstrate aggressive tumour biology that may explain worse clinical outcomes.

## Teaser

Worse genetic profile in tail

## Introduction

Pancreatic ductal adenocarcinoma (PDAC) is expected to become the third leading cause of cancer-related death in Western societies, and to overtake breast cancer for the first time in 2017^1^. The 5-year survival rate, almost unchanged in 50 years, remains less than 10 per cent^1^. Surgical resection is the only chance of cure, with chemotherapy adding modest benefit to overall survival^2–4^. Early recurrence remains a major clinical concern for patients undergoing pancreatectomy, and accurate preoperative prognostication is currently a challenge.

Numerous studies^5–9^ have demonstrated meaningful differences in outcome of pancreatic cancers located in the head, compared with those of the body and tail. Approximately 15 per cent of PDACs occur in the body and tail, and differences in outcome have been largely attributed to late presentation in comparison with tumours of the pancreatic head^5–7,9^. Tumours of the head and uncinate process often present with jaundice, and are therefore thought to present earlier in the disease process. Body and tail pancreatic cancer usually presents with weight loss and pain, symptoms more in keeping with advanced disease^7^. Yet, previous data suggest that TNM stage at presentation is not significantly different between the two tumour locations^10^.

Recent large-scale sequencing studies^11–16^ have demonstrated that PDAC harbours significant interpatient genomic heterogeneity. Apart from a few well known mutations that are currently not targetable (*KRAS, TP53, CDKN2A* and the loss of *SMAD4*), most genetic aberrations occur at low frequency (10 per cent or less). Whole-genome sequencing of 100 resected PDACs demonstrated unique structural variation subtypes based on chromosomal rearrangement numbers and patterns that appear to predict response to platinum-based chemotherapy in a ‘synthetic lethality’ manner^13^. Additionally, integrated genomic analyses^14–16^ revealed distinct molecular subtypes of PDAC based on transcriptomic profiles that correspond to clinical outcomes. Bailey and colleagues^14^ described a poor prognostic ‘squamous’ subtype that is enriched for histopathological adenosquamous tumours, *TP53* mutations^17^, and gene programmes associated with inflammation, hypoxia response, metabolic reprogramming, MYC pathway activation and transforming growth factor β signalling^14^. The squamous subtype was enriched for gene programmes that are common in squamous-like tumours of breast, bladder, lung, and head and neck cancer^18^; and characterized by hypermethylation and downregulation of genes involved in pancreatic endodermal differentiation (*PDX1, MNX1, GATA6, HNF1B*)^14^. In addition, the squamous subtype was enriched for mutations and loss of key epigenetic regulators (such as *KDM6A*) and this may contribute to the loss of endodermal properties of these tumours^14^. Expression patterns of immune cell populations within the tumour microenvironment demonstrated unique differences between transcriptomic subtypes, with evidence of immune avoidance in the squamous subtype^14^.

The molecular pathology of PDAC has recently been studied intensely, yet the exact genetic and molecular differences between PDACs of the head and those of the body and tail have not been fully elucidated. Therefore, the aim of this study was to explore and define the genomic and transcriptomic differences between head and body/tail PDAC based on a large integrated genomic analysis of PDAC^14^.

## Methods

Patients were recruited prospectively through the Australian Pancreatic Cancer Genome Initiative (APGI) as part of the International Cancer Genome Consortium (ICGC). Informed consent and human research ethics approvals were obtained from each contributing clinical centre (*Appendix S1*, supporting information). Contributing patients were restricted to those with resectable, chemotherapy- and radiotherapy-naïve PDAC, who underwent either Whipple's pancreatoduodenectomy, or total or distal pancreatectomy. Following surgical resection, histopathological analysis was performed by a pancreatic pathologist. Specimens with macroscopic evidence of tumour were snap-frozen as a source of tumour DNA, along with samples of duodenum, stomach or spleen as a source of germline DNA. Standard histopathological processing was undertaken, and a diagnosis of PDAC was confirmed independently by two consultant pathologists with a specialist interest in pancreatic cancer. Fresh-frozen tumour samples underwent full-face cryosection to confirm the presence of tumour and to estimate epithelial cellularity. Macrodissection was performed before DNA and RNA extraction to enrich for tumour epithelium. DNA and RNA extraction was carried out using the AllPrep Kit (Qiagen, Hilden, Germany) according to the manufacturer's protocol.

### Data analysis

Whole-exome and/or whole-genome and RNA sequencing was performed and analysed as described previously^12–14^. Briefly, genome sequence data were aligned and mapped using the Genome Reference Consortium GRCh37 assembly, and the Burrows–Wheeler alignment tool^19^. Single-nucleotide substitutions and insertions/deletions, structural variations, copy number analysis, mutational signature and RNA sequencing analysis was carried out^12–14^. Hierarchical clustering, gene set and pathway enrichment analysis was performed using the R package (R Project for Statistical Computing, Vienna, Austria)^14^.

### Statistical analysis

Disease-specific survival was used for all survival analyses. Median survival was estimated by the Kaplan–Meier method, and differences tested using the log rank test^20^. *P* < 0·050 was considered statistically significant. Clinicopathological variables with *P* < 0·100 on log rank testing were entered into a Cox proportional hazards multivariable analysis^21^. Statistical analysis was performed using SPSS® version 22.0 (IBM, Armonk, New York, USA) and R 3.3.1.

## Results

Patient characteristics are summarized in *Table 1* and *Table S1* (supporting information). In total 518 patients with detailed clinical and pathological data were identified; 421 PDACs underwent DNA sequencing, consisting of 179 whole genomes and 242 whole exomes. Ninety-six patients underwent whole-transcriptome RNA sequencing and another 266 underwent transcriptomic characterization based on microarray gene expression analysis owing to lower tumour epithelial content, of whom 262 had survival data available for analysis (*Table S1*, supporting information).

**Table 1:** Characteristics of patients in the Australian Pancreatic Cancer Genome Initiative cohort who underwent resection of pancreatic ductal adenocarcinoma according to tumour location

	Head			Body and tail		
	No. of patients^*^ (*n* = 426)	Median DSS (months)	*P* ^‡^	No. of patients^*^ (*n* = 92)	Median DSS (months)	*P* ^‡^
Age (years)^†^	68·0 (28·0–88·0)			70·5 (28·0–86·0)		
Mean	66·5			67·8		
Sex ratio (M : F)	213 : 213		0·904	45 : 47		0·168
Outcome						
Follow-up (months)^†^	48 (18–164)			45 (32–136)		
Died						
Pancreatic cancer	196 (46·0)			47 (51)		
Other	10 (2·3)			5 (5)		
Unknown	0 (0)			0 (0)		
Alive	215 (50·5)			40 (43)		
Lost to follow-up	5 (1·2)			0 (0)		
Tumour stage			< 0·001			0·018
I	24 (5·6)	56·6		8 (9)	51·9	
II	400 (93·9)	21·0		74 (80)	13·0	
III	1 (0·2)	20·0		1 (1)	21·0	
IV	1 (0·2)	5·7		9 (10)	7·6	
T category			0·117			0·142
T1	16 (3·8)	31·0		5 (5)	73·0	
T2	34 (8·0)	32·0		14 (15)	15·8	
T3	375 (88·0)	21·0		72 (78)	11·6	
T4	1 (0·2)	20·0		1 (1)	21·0	
N category			0·004			0·724
N0	134 (31·5)	25·2		35 (39)	12·0	
N1	292 (68·5)	20·7		55 (61)	13·0	
Tumour grade			0·012			0·903
I	32 (7·5)	38·1		9 (10)	15·8	
II	283 (66·6)	23·0		57 (63)	12·1	
III	107 (25·2)	17·0		23 (25)	13·0	
IV	3 (0·7)	13·0		2 (2)	9·0	
Tumour size (mm)			0·005			0·004
≤ 20	92 (21·6)	32·0		9 (10)	72·6	
> 20	333 (78·4)	19·0		80 (87)	12·0	
Surgical margins (R0 = 0 mm)			< 0·001			0·152
Clear	285 (66·9)	25·2		53 (58)	14·0	
Involved	141 (33·1)	16·7		39 (42)	11·4	
Perineural invasion			0·020			0·556
No	94 (22·5)	29·7		20 (22)	13·0	
Yes	324 (77·5)	20·0		69 (78)	12·1	
Vascular invasion			0·002			0·045
No	193 (46·8)	25·0		41 (47)	15·4	
Yes	219 (53·2)	19·4		46 (53)	11·6	
Adjuvant chemotherapy			< 0·001			0·013
< 3 cycles	249 (58·7)	16·5		61 (66)	9·3	
≥ 3 cycles	175 (41·3)	29·9		31 (34)	17·0	

* With percentages in parentheses unless indicated otherwise;

† values are median (range). Data were missing for some variables. DSS, disease-specific survival.

‡ Log rank test.

### Prognosis after pancreatectomy in relation to tumour location

In the APGI cohort, the majority of tumours (426, 82·2 per cent) were located in the head of the pancreas and 92 (17·8 per cent) presented with body and tail pancreatic cancer. There was no difference in patient demographics between those presenting with head *versus* body/tail tumours (*Table 1*). Body and tail pancreatic cancers were more likely to be of lower pathological T category (T1–2) (21 *versus* 11·7 per cent; *P* = 0·021), yet significantly larger at the time of resection (*P* = 0·007) (*Table S2*, supporting information). Although there was no discernible difference in well known prognostic pathological variables between the two groups (*Table S2*, supporting information), survival was significantly worse in patients presenting with body and tail tumours (median survival 12·1 *versus* 22·0 months; *P* = 0·001) (*Fig. S1A*, supporting information). This remained significant in multivariable analysis (hazard ratio 1·72, 95 per cent c.i. 1·31 to 2·26; *P* < 0·001) (*Table S3*, supporting information).

### Association between body and tail cancers and squamous subtype

Cancers of the body and tail of pancreas co-segregated with the squamous subtype of pancreatic cancer, both among patients who underwent whole-transcriptome sequencing (96 patients; *P* = 0·033) and those who underwent mRNA microarray sequencing (non-redundant set, 266 patients; *P* < 0·001) (*Table 2*). There was no association between tumour location and chromosomal structural variation subtype (*P* = 0·211) (*Table S4*, supporting information). However, body and tail pancreatic cancer was associated with a *BRCA* mutational signature (Catalogue of Mutational Processes in Cancer (COSMIC)) (*P* = 0·025) (*Table S4*, supporting information). Based on the frequency of mutations per megabase, COSMIC mutational signature 8 (unknown aetiology) was associated with tumour location (*P* = 0·002), but not signatures associated with loss of mismatch repair status (*P* = 0·619), oesophageal cancer (COSMIC mutational signature 17) (*P* = 0·976), deamination (*P* = 0·287) and apolipoprotein B mRNA editing enzyme, catalytic polypeptide-like (APOBEC) (*P* = 0·301).

**Table 2:** Association between tumour location and Bailey subtype

	Non-squamous	Squamous	*P* ^*^
RNA sequencing cohort (*n* = 96)			0·033
Head	60 (85)	16 (64)	
Body/tail	11 (15)	9 (36)	
Microarray cohort (*n* = 266)			< 0·001
Head	163 (88·6)	57 (70)	
Body/tail	21 (11·4)	25 (30)	

Values in parentheses are percentages.

* χ^2^ test.

### Association between squamous subtype, tumour grade and recurrence

In view of the significant association between the poor prognostic squamous subtype and body and tail pancreatic cancer, a detailed clinical and pathological analysis of the squamous subtype was undertaken in 96 patients who underwent whole-transcriptome sequencing as part of the ICGC project. This demonstrated a significant association with poor tumour differentiation and higher tumour grade (*P* < 0·001), but not with pathological T category (*P* = 0·467), node status (*P* = 0·520) or surgical margin status (*P* = 0·615) (*Table 3*). These findings were recapitulated in the microarray set (*Table S5*, supporting information).

**Table 3:** Association between clinicopathological variables, tumour recurrence patterns and pancreatic adenocarcinoma subtype based on RNA sequencing set

	Non-squamous (*n* = 71)	Squamous (*n* = 25)	*P* ^†^
T category			0·467
T1–2	11 (16)	3 (12)	
T3–4	59 (84)	22 (88)	
N category			0·520
N0	25 (36)	8 (33)	
N1	45 (64)	16 (67)	
Tumour grade/differentiation			< 0·001
I/II	52 (74)	5 (22)	
III/IV	18 (26)	18 (78)	
Perineural invasion			0·027
No	9 (13)	8 (35)	
Yes	59 (87)	15 (65)	
Vascular invasion			0·256
No	31 (46)	8 (35)	
Yes	37 (54)	15 (65)	
Tumour size (mm)			0·185
≤ 20	10 (14)	1 (4)	
> 20	60 (86)	22 (96)	
Surgical margin			0·615
Negative	58 (83)	20 (83)	
Positive	12 (17)	4 (17)	
Histological subtype			0·014
IPMN with invasion	13 (18)	0 (0)	
PDAC – NOS	58 (82)	25 (100)	
Local recurrence^*^			0·194
No	37 (90)	20 (100)	
Yes	4 (10)	0 (0)	
Liver recurrence^*^			0·002
No	25 (56)	3 (15)	
Yes	20 (44)	17 (85)	
Lung recurrence^*^			0·345
No	33 (73)	13 (65)	
Yes	12 (27)	7 (35)	
Lung recurrence (not liver)^*^			0·432
No	38 (84)	18 (90)	
Yes	7 (16)	2 (10)	
Non-liver distant recurrence^*^			0·040
No	27 (66)	18 (90)	
Yes	14 (34)	2 (10)	

Values in parentheses are percentages. Data were missing for some variables.

* Recurrence data shown only for patients who developed any recurrence during the study interval.

† χ^2^ test.

IPMN, intraductal papillary mucinous neoplasm; PDAC, pancreatic ductal adenocarcinoma; NOS, not otherwise specified.

The squamous subtype was associated significantly with liver recurrence (*P* = 0·002) (*Table 3*), and liver recurrence was associated with significantly worse survival compared with local and other distant recurrence patterns (median survival 13·6 *versus* 20·0 months respectively; *P* < 0·001) (*Fig. S1B*, supporting information), regardless of primary tumour location. Furthermore, all 20 patients with squamous tumours that had developed recurrence during follow-up died from distant metastatic disease (*Table 3*). There was no association between lung recurrence and transcriptomic subtypes (*P* = 0·345).

### Prognosis in relation to tumour location and squamous subtype

**Fig. 1:**
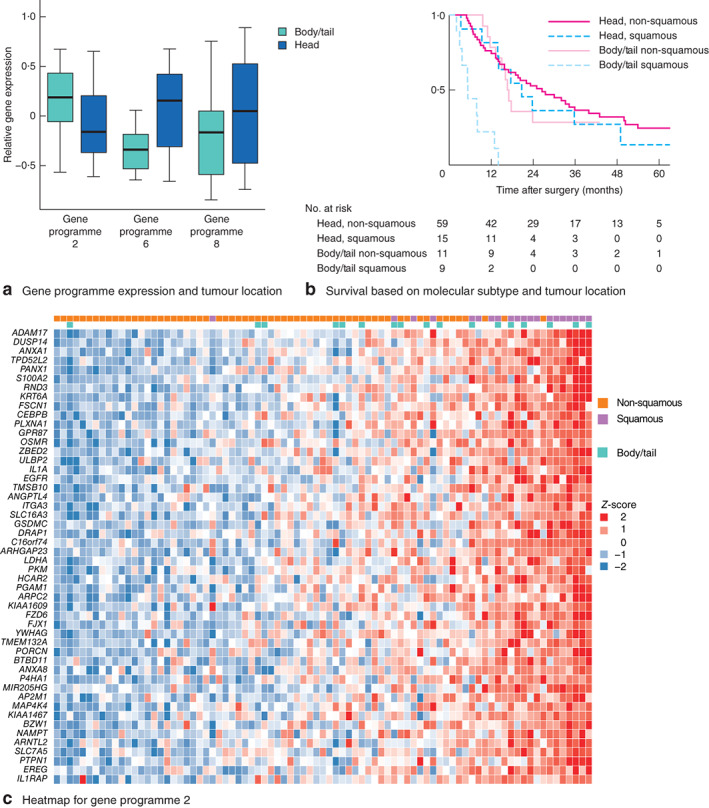
**a** Association between expression of gene programmes 2, 6 and 8 and tumour location. Median values (bold line), i.q.r. (box) and range (error bars) are shown. ^*^P < 0·050 (Kruskal–Wallis test). **b** Kaplan–Meier curve showing survival according to Bailey subtype and tumour location in 94 patients from the RNA sequencing set. *P* = 0·010 (log rank test). **c** Heatmap of relative gene expression within gene programme 2 demonstrating strong correlation between squamous subtype, body and tail tumours, and gene programme 2

Kaplan–Meier survival analysis demonstrated that patients with the squamous subtype of PDAC had a significantly worse prognosis in the RNAseq set (median survival 13·3 months *versus* 23·7 months in those with non-squamous tumours; *P* = 0·010) (*Fig. S1C*, supporting information). Resected tumours were segregated into tumour location and transcriptomic subtype, as defined in Bailey *et al*.^14^, to assess the difference in prognosis in squamous tumours of the head or body and tail. Patients who had squamous tumours of the body and tail had an extremely poor survival compared with the rest of the cohort (median survival 5·2 *versus* 22·0 months; *P* < 0·001) (*Fig. 1b*). These findings were recapitulated in the microarray set (median survival 25·0, 18·4, 15·9 and 11·5 months among patients with non-squamous tumours of the head, squamous tumours of the head, non-squamous tumours of the body/tail and squamous tumours of the body/tail respectively; *P* = 0·001) (*Fig. S1D*, supporting information).

The prognostic value of mutational signatures was assessed against the APGI cohort in 167 patients who underwent whole-genome sequencing and had sufficient clinical data available for analysis. There was no relationship between mutational signatures associated with loss of mismatch repair (*P* = 0·573), signature 8 (unknown aetiology) (*P* = 0·227), signature 17 (oesophageal cancer) (*P* = 0·639), deamination (*P* = 0·716), APOBEC (*P* = 0·899) or *BRCA* (*P* = 0·575) and survival in patients with resected PDAC.

### Association between body and tail pancreatic cancer and molecular features of aggressive disease

An in-depth analysis of tumour location in relation to gene programmes that define the Bailey subtypes was undertaken. Body and tail pancreatic cancer was significantly associated with gene networks involved in epithelial-to-mesenchymal transition (EMT), inflammation, hypoxia response, metabolic reprogramming, TP63 expression and squamous differentiation (gene programme 2) (*Fig. 1a*)^14^. Conversely, head tumours were enriched for gene programmes 6 and 8, which are associated with B cell and CD8-positive T cell signalling respectively^14^ (*Fig. 1*; *Table S6*, and *Figs S2* and *S3*, supporting information). Body and tail pancreatic cancer exhibited immune signatures corresponding to low dendritic cell infiltrate (*P* = 0·005), low co-stimulation of antigen-presenting cells (*P* = 0·041) and a low type II interferon response (*P* = 0·002) (*Table S6*, supporting information). These findings suggest that, relative to pancreatic head cancer, body and tail tumours are associated with more aggressive disease biology and potentially exhibit a dampened antitumour immune response and increased immune avoidance.

## Discussion

Clinical outcomes for body and tail pancreatic cancer appear to be significantly worse in both the resectable and advanced disease stages. The present study has demonstrated distinct molecular differences between resectable PDAC from the head and body/tail. Body and tail pancreatic cancer is associated with the squamous subtype of pancreatic cancer and enriched for gene programmes associated with inflammation, EMT and potential immune avoidance mechanisms.

**Fig. 2:**
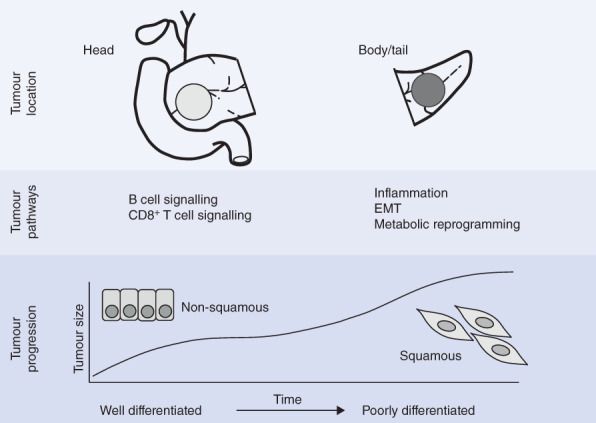
Graphical representation of the association between pancreatic adenocarcinoma location and differential transcriptional networks in the Australian Pancreatic Cancer Genome Initiative cohort. A potential theory of subtype evolution suggests that tumour size increases along the molecular clock, associated with dedifferentiation from pancreatic progenitor-like to squamous-like. EMT, epithelial-to-mesenchymal transition

The temporal sequence of genomic and epigenomic events leading to the progression to different PDAC subtypes has yet to be fully elucidated. However, the squamous subtype appears to be more advanced on the molecular clock than other subtypes, and this may reflect an additional level of genomic instability, owing to the accumulation of DNA damage, and molecular events that contribute to the unique transcriptome of these tumours^14^. Previous studies^14–16^ have found that these tumours are enriched for epigenetic events leading to hypermethylation and downregulation of genes involved in pancreatic development, and enriched for molecular drivers of EMT. The present results suggest that body and tail pancreatic cancer is more likely to be of a squamous subtype, suggesting that these are biologically more aggressive at the time of diagnosis, or surgical resection, than cancers of the pancreatic head. The results also suggest that the squamous subtype of PDAC is associated predominantly with liver recurrence; this may in part explain the worse prognosis of patients with liver metastases compared with those with metastases at other distant sites^22^. In this cohort, none of the intraductal papillary mucinous neoplasm-related PDACs were classified as the squamous subtype (*Table 3*), potentially revealing a different carcinogenesis pathway for the squamous subtype^14^. The squamous subtype correlates with increased size and poor tumour differentiation, which may reflect tumours that present at a later stage, or have an accelerated dedifferentiation pathway (*Fig. 2*).

Transcriptomic subtyping of PDAC remains a debated topic, and it has recently been suggested that there is significant overlap between the squamous subtype described by Bailey *et al*.^14^, and the basal^16^ and quasi-mesenchymal^15^ classifications. The role of stromal factors and immune infiltrate remains crucial to tumour growth and response to therapy, and is likely to remain a crucial aspect of molecular subtyping in PDAC^14,16^. As a result, the implications of tumour cellularity on transcriptomic analysis remain a priority area of research. However, several studies^23–25^ have demonstrated that epithelial cell lines of PDAC recapitulate published transcriptomic subtypes, including transcripts native to pancreatic exocrine and endocrine cells defining an aberrantly differentiated endocrine exocrine subtype (ADEX).

Genes involved in inflammation, EMT and invasion are enriched in body and tail pancreatic cancer; these molecular factors are known to be associated with poor prognosis. Levels of mRNA transcripts of calcium-binding protein S100A2, which accelerates tumour invasion, are greater in body and tail pancreatic cancer than in cancers of the pancreatic head, and this is one of the most differentially expressed genes in gene programme 2 (*Fig. 1*). High S100A2 expression has been shown previously to be highly prognostic in PDAC, and forms a key molecular predictor of early recurrence in a preoperative molecular nomogram for operable PDAC (S. B. Dreyer, M. Pinese, N. B. Jamieson, C. J. McKay, A.V. Biankin, D. K. Chang *et al*., unpublished data)^26,27^. In the RNAseq cohort here, patients with squamous subtype body and tail pancreatic cancer had extremely poor survival (median 5·2 months), in comparison with the rest of the resected cohort. This suggests that such patients may be better treated with a neoadjuvant approach as occult metastatic disease may manifest over this period, and avoid futile major surgery. Because of the molecular features of aggressive disease associated with body and tail pancreatic cancer, it could also be argued that, until molecular markers of early recurrence are better defined, all body and tail tumours are better served with a neoadjuvant approach to identify patients whose tumours are likely to recur early.

It has been demonstrated here that head tumours are relatively enriched for B cell signalling (gene programme 6) and this has been shown to be associated with a better prognosis^14^. Similarly, body and tail pancreatic cancer lacks CD8-positive T cell signalling (gene programme 8), suggesting an immunosuppressive tumour microenvironment. This may well reflect an increase in myeloid cell infiltration and tumour-associated macrophage-related immunosuppression and inflammation^28^. In-depth analysis of the tumour microenvironment may reveal potential targets for novel immunotherapy agents in body and tail pancreatic cancer, including the immune checkpoint and myeloid signalling axes^28^.

It remains to be determined whether body and tail pancreatic cancer presents at a later stage of tumour evolution, or whether these tumours are biologically different and more aggressive from the outset. However, some of the present findings seem to reflect the relatively late presentation of body and tail pancreatic cancer at a more advanced stage, both clinically and molecularly (*Fig. 2*). First, tumours of the body and tail are larger, which may reflect a biologically older tumour. Second, body and tail pancreatic cancers correlate with molecular features that are driven by epigenetic events associated with chromosomal instability and epigenetic events that may drive intratumoral heterogeneity^29,30^. The exact sequence of these events in tumorigenesis and progression has yet to be elucidated, but may be associated with a later stage of disease evolution.

Identifying patients who will benefit from procedures with high morbidity rates such as pancreatectomy is an important task for surgeons. Personalized medicine platforms, such as Precision Panc^31^, will allow clinicians and scientists to correlate molecular profiles with clinicopathological outcomes, as well as define and refine molecular subgroups that respond to personalized treatment regimens, including surgery. Well designed clinical trials, particularly in the operable and neoadjuvant setting, will allow detailed study of the temporal and spatial clonal evolution of PDAC, and may shed light on the relationship between disease progression and the molecular timeline of tumours. This will contribute to an expanding knowledge bank of molecular and clinical data, acquired from multiple initiatives globally, and will further delineate the relationship between tumour location, stage at presentation and the molecular features of PDAC.

## Supplementary Material

bjs10772-sup-0001-AppendixS1 **Appendix S1** Ethical statement and ethics approval
**Table S1** Patient characteristics for the APGI mRNA Sequenced Cohort (subtyped according to micro-array sequencing). Survival analysis for *n* = 262 with available outcome data during study period.
**Table S2** Clinicopathological variables and tumour location in APGI cohort
**Table S3** Multivariate models for APGI Cohort
**Table S4** Tumour location and association with SV subtypes and mutational signatures (WGS set *n* = 179)
**Table S5** Association between clinicopathological variables and PDAC subtype based on mRNA micro-array set (*n* = 266)
**Table S6** Tumour location and association with immune signatures of PDAC in RNAseq set (*n* = 96)
**Fig. S1** Kaplan–Meier survival curves for A) tumour location, B) recurrence pattern, C) Bailey subtype and D) Bailey subtype (micro-array set) and tumour location
**Fig. S2** Heatmap of relative gene expression of Gene Program 6. Corresponding Bailey subtype and tumour location indicates correlation between squamous subtype, body and tail tumours and low expression within GP6
**Fig. S3** Heatmap of relative gene expression of Gene Program 8. Corresponding Bailey subtype and tumour location indicates correlation between body and tail tumours and low expression within GP8
