# Supplementary material for: Defining the molecular pathology of pancreatic body and tail adenocarcinoma
Source: Br J Surg. 2018 Jan 17;105(2):e183–91. doi: 10.1002/bjs.10772 (PMC5817249; doi:10.1002/bjs.10772)
Supplement: bjs10772-sup-0001-AppendixS1 — Appendix S1 Ethical statement and ethics approval Table S1 Patient characteristics for the APGI mRNA Sequenced Cohort (subtyped according to micro-array sequencing). Survival analysis for n = 262 with available outcome data during study period. Table S2 Clinicopathological variables and tumour location in APGI cohort Table S3 Multivariate models for APGI Cohort Table S4 Tumour location and association with SV subtypes and mutational signatures (WGS set n = 179) Table S5 Association between clinicopathological variables and PDAC subtype based on mRNA micro-array set (n = 266) Table S6 Tumour location and association with immune signatures of PDAC in RNAseq set (n = 96) Fig. S1 Kaplan–Meier survival curves for A) tumour location, B) recurrence pattern, C) Bailey subtype and D) Bailey subtype (micro-array set) and tumour location Fig. S2 Heatmap of relative gene expression of Gene Program 6. Corresponding Bailey subtype and tumour location indicates correlation between squamous subtype, body and tail tumours and low expression within GP6 Fig. S3 Heatmap of relative gene expression of Gene Program 8. Corresponding Bailey subtype and tumour location indicates correlation between body and tail tumours and low expression within GP8 [file bjs10772-sup-0001-appendixs1.docx]

**Defining the molecular pathology of pancreatic body and tail adenocarcinoma**

S. B. Dreyer, N. B. Jamieson, R. Upstill-Goddard, P. J. Bailey, C. J. McKay, A. V. Biankin, D. K. Chang and members of the Australian Pancreatic Cancer Genome Initiative

**Appendix S1** Ethical statement and ethics approval

**Ethical Statement**

Ethical approval for the acquisition of data and biological material was obtained from the Human Research Ethics Committee at each participating institution (Data Supplement).

**Ethics approval numbers**

**APGI:**

- Sydney South West Area Health Service Human Research Ethics Committee, Western Zone, protocol number 2006/54
- Sydney Local Health District Human Research Ethics Committee, protocol number X11-0220
- Northern Sydney Central Coast Health Human Research Ethics Committee, protocol number 0612-251M
- Sydney West Area Health Service Human Research Ethics Committee (Westmead Campus), protocol number HREC2002/3/4.19
- South East Sydney Illawarra Area Health, Northern Hospital Network HREC- protocol number 05/321
- South East Sydney Illawarra Area Health HREC- Southern Section, protocol number 05/54

**Table S1** Patient characteristics for the APGI mRNA Sequenced Cohort (subtyped according to micro-array sequencing). Survival analysis for *n* = 262 with available outcome data during study period.

|  | **Non-squamous** | | | **Squamous** | | |
| --- | --- | --- | --- | --- | --- | --- |
| **Variables** | ***n* = 184**  **No. (%)** | **Median DSS**  **(months)** | ***P* value**  **(Logrank)** | ***n* = 82**  **No. (%)** | **Median DSS**  **(months)** | ***P* value**  **(Logrank)** |
| **Sex** |  |  |  |  |  |  |
| Male | 92 (51.1) | 19.6 | 0.079 | 47 (57.3) | 13.8 | 0.574 |
| Female | 88 (48.9) | 29.0 |  | 35 (42.7) | 16.6 |  |
| **Age (years)** |  |  |  |  |  |  |
| Mean | 66.7 |  |  | 66.7 |  |  |
| Median | 68 |  |  | 67 |  |  |
| Range | 34 - 90 |  |  | 36 – 90 |  |  |
| **Outcome** |  |  |  |  |  |  |
| Follow-up (months) | 7.5 – 81.0 |  |  | 2.8 – 79.0 |  |  |
| Median follow-up(months) | 44.0 |  |  | 40.5 |  |  |
| Death PC | 124 (68.1) |  |  | 63 (76.9) |  |  |
| Death other | 10 (5.5) |  |  | 7 (8.5) |  |  |
| Death Unknown | 0 (0.0) |  |  | 0 (0.0) |  |  |
| Alive | 48 (26.4) |  |  | 12 (14.6) |  |  |
| Lost to FU | 0 (0.0) |  |  | 0 |  |  |
| **Stage** |  |  |  |  |  |  |
| I | 11 (7.4) | 9.4 | 0.016 | 8 (9.8) | 73.0 | 0.041 |
| II | 164 (91.6) | 25.0 |  | 67 (81.7) | 14.0 |  |
| III | 0 (0.0) | - |  | 1 (1.2) | 14.1 |  |
| IV | 4 (2.2) | 13.0 |  | 6 (7.3) | 14.9 |  |
| **T Stage** |  |  |  |  |  |  |
| T1 | 5 (2.8) | - | 0.412 | 1 (1.2) | 73.0 | 0.091 |
| T2 | 28 (15.6) | 19.3 |  | 12 (14.6) | 23.7 |  |
| T3 | 147 (81.6) | 24.0 |  | 66 (80.5) | 13.8 |  |
| T4 | 0 (0.0) | - |  | 3 (3.7) | 14.9 |  |
| **N Stage** |  |  |  |  |  |  |
| N0 | 44 (24.4) | 30.0 | 0.048 | 18 (22.0) | 32.0 | 0.026 |
| N1 | 136 (75.6) | 20.7 |  | 64 (78.0) | 14.9 |  |
| **Grade** |  |  |  |  |  |  |
| I | 16 (8.9) | 33.0 | 0.018 | 0 (0.0) | - | 0.607 |
| II | 118 (65.9) | 25.0 |  | 37 (45.7) | 17.0 |  |
| III | 40 (22.3) | 18.3 |  | 44 (54.3) | 11.9 |  |
| IV | 5 (2.8) | 13.3 |  | 0 (0.0) | - |  |
| **Tumour size** |  |  |  |  |  |  |
| ≤ 20mm | 21 (11.7) | 37.5 | 0.290 | 7 (8.6) | 73.0 | 0.085 |
| > 20mm | 159 (88.3) | 20.7 |  | 74 (91.4) | 14.1 |  |
| **Margins (R0 = 0mm)** |  |  |  |  |  |  |
| Clear | 135 (75.0) | 27.0 | 0.005 | 55 (67.1) | 16.6 | 0.225 |
| Involved | 45 (25.0) | 17.4 |  | 27 (32.9) | 12.1 |  |
| **Tumour Location** |  |  |  |  |  |  |
| Head | 159 (88.3) | 25.0 | 0.197 | 57 (69.5) | 18.4 | 0.032 |
| Body / Tail | 21 (11.7) | 17.6 |  | 25 (30.5) | 11.5 |  |
| **Perineural Invasion** |  |  |  |  |  |  |
| Negative | 29 (16.3) | 40.0 | 0.008 | 17 (21.3) | 23.0 | 0.021 |
| Positive | 149 (83.7) | 20.0 |  | 63 (78.7) | 13.6 |  |
| **Vascular Invasion** |  |  |  |  |  |  |
| Negative | 73 (41.7) | 33.3 | 0.003 | 26 (32.5) | 23.0 | 0.002 |
| Positive | 102 (58.3) | 19.0 |  | 54 (67.5) | 13.6 |  |

**Table S2** Clinicopathological variables and tumour location in APGI cohort

|  | | | |
| --- | --- | --- | --- |
|  | **Head** | **Body / Tail** | ***P*-value**  **(Chi-square)** |
| **T - stage**  T I / II  T III / IV | 50 (11.7%)  376 (88.3%) | 19 (20.7%)  73 (79.3%) | 0.021 |
| **N - stage**  N0  N1 | 134 (31.5%)  292 (68.5%) | 35 (38.9%)  55 (61.1%) | 0.108 |
| **Grade**  I / II  III / IV | 303 (73.4%)  110 (26.6%) | 62 (71.3%)  25 (28.7%) | 0.390 |
| **Perineural Invasion**  Negative  Positive | 94 (22.5%)  324 (77.5%) | 20 (22.5%)  69 (77.5%) | 0.561 |
| **Vascular space invasion**  Negative  Positive | 193 (46.8%)  219 (53.2%) | 41 (47.1%)  46 (52.9%) | 0.527 |
| **Size**  < 20mm  ≥ 20mm | 92 (21.6%)  333 (78.4%) | 9 (10.1%)  80 (89.9%) | 0.007 |
| **Margin (R0 = 0mm)**  R0  R1 | 285 (66.9%)  141 (33.1%) | 53 (57.6%)  39 (42.4%) | 0.059 |
| **Adjuvant Chemotherapy**  < 3 cycles  ≥ 3 cycles | 249 (58.7%)  175 (41.3%) | 61 (66.3%)  31 (33.7%) | 0.109 |

**Table S3** Multivariate models for APGI Cohort

|  | | | |
| --- | --- | --- | --- |
|  | **Variable** | **Hazard Ratio (95% CI)** | ***P* Value** |
| **Clinicopathological variables and Tumour location**  **(*n* = 465)** | Differentiation (poor) | 1.32 (1.04 – 1.67) | *0.023* |
|  | T Stage (T3/4) | 1.35 (0.96 – 1.89) | 0.088 |
|  | Lymph Node Involvement | 1.13 (0.89 – 1.44) | 0.307 |
|  | Margin Involvement (Positive) | 1.73 (1.38 – 2.17) | < 0.001 |
|  | Tumor Location (Body/Tail) | 1.70 (1.29 0 2.22) | < 0.001 |
|  | Perineural Invasion (Positive) | 1.15 (0.88 – 1.49) | 0.305 |
|  | Vascular Invasion (Positive) | 1.23 (0.97 – 1.55) | 0.085 |
|  | Differentiation (poor) | 1.32 (1.04 – 1.67) | 0.024 |
|  | T Stage (T3/4) | 1.37 (0.98 – 1.93) | 0.068 |
|  | Margin Involvement (Positive) | 1.75 (1.40 – 2.18) | <0.001 |
|  | Tumor Location (Body/Tail) | 1.71 (1.31 – 2.24) | <0.001 |
|  | Perineural Invasion (Positive) | 1.15 (0.89 – 1.49) | 0.298 |
|  | Vascular Invasion (Positive) | 1.27 (0.89 – 1.49) | 0.298 |
|  | Differentiation (poor) | 1.30 (1.03 – 1.65) | 0.030 |
|  | T Stage (T3/4) | 1.37 (0.97 – 1.92) | 0.070 |
|  | Margin Involvement (Positive) | 1.78 (1.43 – 2.22) | <0.001 |
|  | **Tumor Location (Body/Tail)** | **1.72 (1.31 – 2.26)** | **<0.001** |
|  | Vascular Invasion (Positive) | 1.30 (1.01 – 1.62) | 0.019 |

**Table S4** Tumour location and association with SV subtypes and mutational signatures (WGS set *n* = 179)

|  | | | |
| --- | --- | --- | --- |
|  | **Head** | **Body / Tail** | ***P*-value**  **(Chi-square)** |
| **SV subtype**  Stable  Focal  Scattered  Unstable | 20 (14.3%)  46 (32.9%)  61 (43.6%)  13 (9.3%) | 5 (15.2%)  5 (15.2%)  20 (60.6%)  3 (9.1%) | 0.211 |
|  | **Mean mutations per MB, *P* calculated by ANOVA table** | | |
| **MMR** | 1.34 | 0.73 | 0.619 |
| **Unknown** | 0.38 | 0.56 | 0.002 |
| **Oesophageal Cancer** | 0.51 | 0.51 | 0.976 |
| **Deamination** | 1.80 | 1.98 | 0.287 |
| **APOBEC** | 0.74 | 0.93 | 0.301 |
| **BRCA** | 1.41 | 2.17 | 0.025 |

**Table S5** Association between clinicopathological variables and PDAC subtype based on mRNA micro-array set (*n* = 266)

|  | | | |
| --- | --- | --- | --- |
|  | **Non-Squamous** | **Squamous** | ***P*-value**  **(Chi-square)** |
| **T - stage**  T I / II  T III / IV | 34 (18.5%)  150 (81.5%) | 13 (15.9%)  69 (84.1%) | 0.370 |
| **N - stage**  N0  N1 | 44 (24.0%)  139 (76.0%) | 18 (22.0%)  64 (78.0%) | 0.419 |
| **Grade / Differentiation**  I / II  III / IV | 135 (73.8%)  48 (26.2%) | 37 (45.7%)  44 (54.3%) | <0.001 |
| **Perineural Invasion**  Negative  Positive | 29 (16.0%)  152 (84%) | 17 (21.3%)  63 (78.8%) | 0.198 |
| **Vascular space invasion**  Negative  Positive | 75 (42.1%)  103 (57.9%) | 26 (32.5%)  54 (67.5%) | 0.091 |
| **Size**  ≤ 20mm  > 20mm | 22 (12.0%)  161 (88.0%) | 7 (8.6%)  74 (91.4%) | 0.281 |
| **Margin**  Negative  Positive | 138 (75.4%)  45 (24.6%) | 55 (67.1%)  27 (32.9%) | 0.104 |

**Table S6** Tumour location and association with immune signatures of PDAC in RNAseq set (*n* = 96)

|  | | | |
| --- | --- | --- | --- |
|  | **Head** | **Body / Tail** | ***P*-value**  **(Chi-square)** |
| **B Cells**  Low  High | 50 (65.8%)  26 (34.2%) | 17 (85%)  3 (15.0%) | 0.078 |
| **CD4 Reg T cells**  Low  High | 50 (65.8%)  26 (34.2%) | 16 (80%)  4 (20%) | 0.172 |
| **CD8 T cells**  Low  High | 49 (64.5%)  27 (35.5%) | 17 (85%)  3 (15%) | 0.064 |
| **Macrophages**  Low  High | 26 (34.2%)  50 (65.8%) | 7 (35%)  13 (65%) | 0.572 |
| **Neutrophils**  Low  High | 55 (72.4%)  21 (27.6%) | 13 (65%)  7 (35%) | 0.349 |
| **Natural Killer Cells**  Low  High | 53 (69.7%)  23 (30.3%) | 14 (70%)  6 (30%) | 0.607 |
| **Dendritic Cells**  Low  High | 49 (64.5%)  27 (35.5%) | 19 (95.0%)  1 (5.0%) | 0.005 |
| **MHC Class 1**  Low  High | 50 (65.8%)  26 (34.2%) | 15 (75%)  5 (25.0%) | 0.309 |
| **Co-Stimulation APC**  Low  High | 47 (61.8%)  29 (38.2%) | 17 (85.0%)  3 (15.0%) | 0.041 |
| **Co-Stimulation T Cells**  Low  High | 53 (69.7%)  23 (30.3%) | 15 (75.0%)  5 (25.0%) | 0.436 |
| **Co-Inhibition APC**  Low  High | 51 (67.1%)  25 (32.9%) | 17 (85.0%)  3 (15.0%) | 0.095 |
| **Co-Inhibition T cells**  Low  High | 26 (34.2%)  50 (65.8%) | 9 (45.0%)  11 (55.0%) | 0.262 |
| **Type I Interferon response**  Low  High | 52 (68.4%)  24 (31.6%) | 14 (70.0%)  6 (30.0%) | 0.562 |
| **Type II Interferon response**  Low  High | 46 (60.5%)  30 (39.5%) | 19 (95.0%)  1 (5.0%) | 0.002 |
| **Cytolytic activity**  Low  High | 52 (68.4%)  24 (31.6%) | 15 (75.0%)  5 (25.0%) | 0.391 |

**
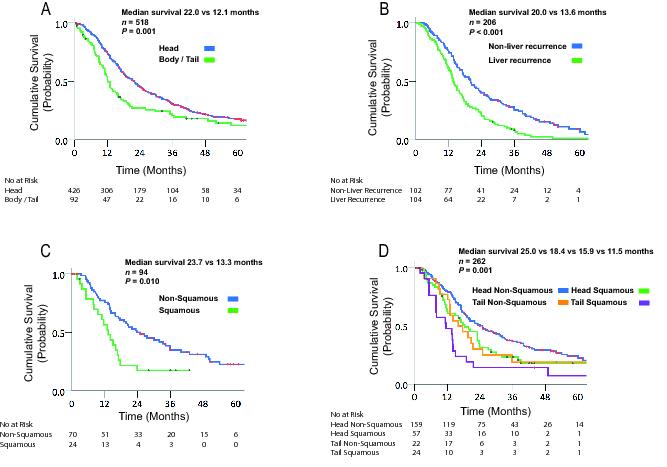
**

**Fig. S1** Kaplan-Meier survival curves for A) tumour location, B) recurrence pattern, C) Bailey subtype and D) Bailey subtype (micro-array set) and tumour location


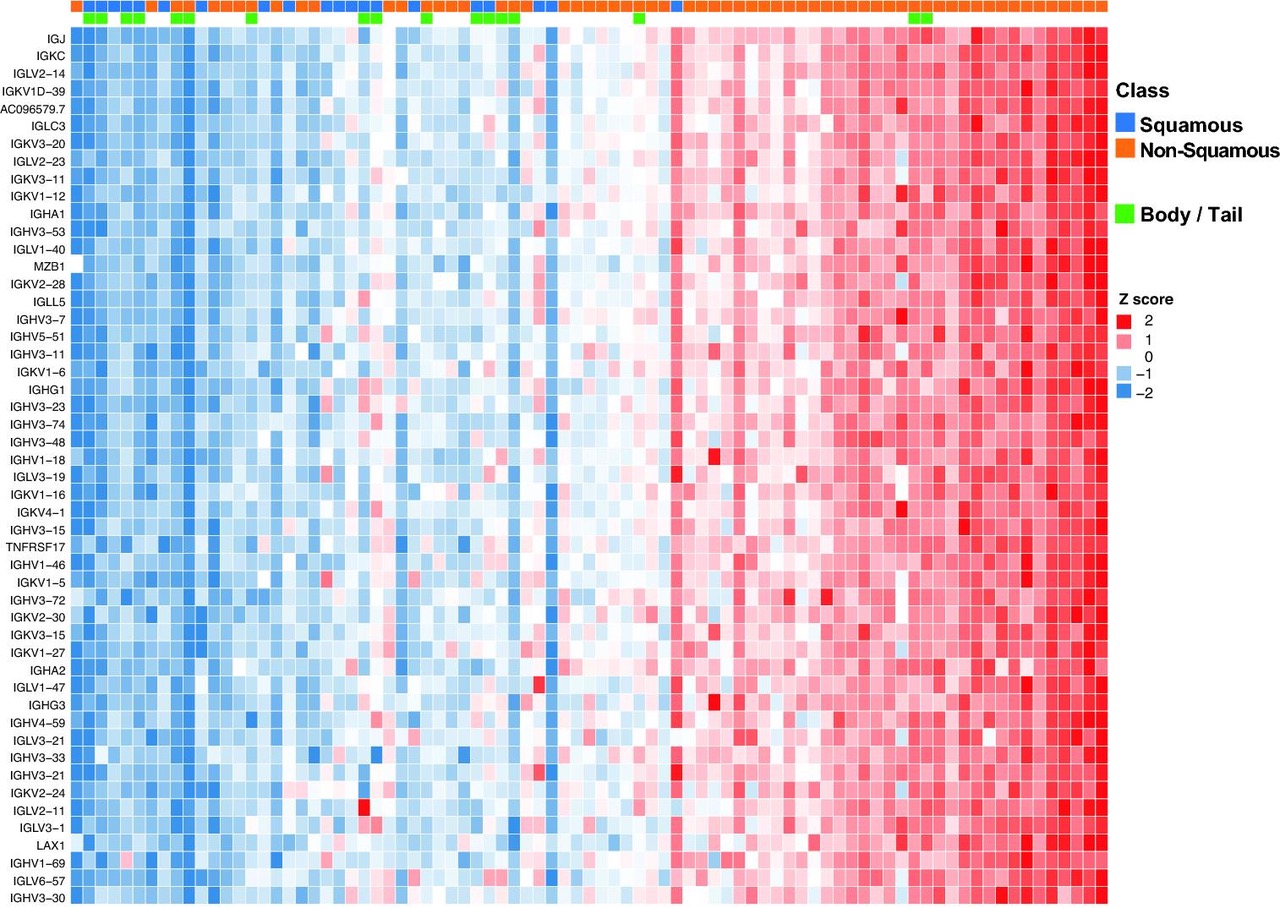


**Fig. S2** Heatmap of relative gene expression of Gene Program 6. Corresponding Bailey subtype and tumour location indicates correlation between squamous subtype, body and tail tumours and low expression within GP6


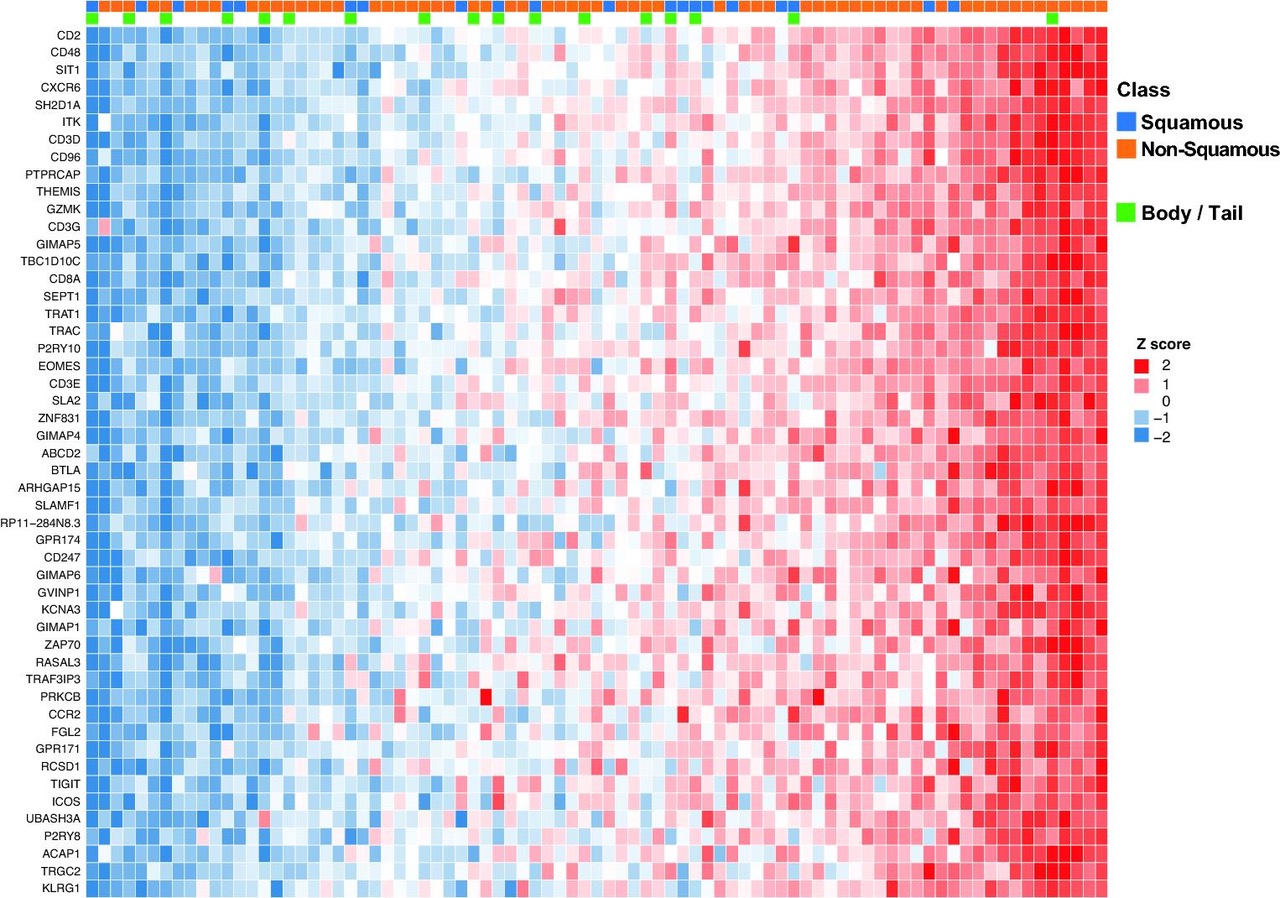


**Fig. S3** Heatmap of relative gene expression of Gene Program 8. Corresponding Bailey subtype and tumour location indicates correlation between body and tail tumours and low expression within GP8
